# Protest from Dr. H. R. Storer

**Published:** 1878-10

**Authors:** 


### Protest from Dr. H. R. Storer.

At the annual meeting of the Massachusetts Medical Society the following protest was received by the President and referred to the appropriate committee without reading:

NEWPORT, R. I., June 12, 1878, 9 A. M.

*To the President of the Massachusetts Medical Society, now in session at Lowell Institute Hall, Boston.*

I had intended to be present to-day in person, but at the last moment my strength has failed. Be kind enough, therefore, to convey to the Society in open meeting my respectful protest against the censure of the Councilors in 1870 for having, as a regular accredited representative of the Gynæcological Society of Boston, presented to the American Medical Association a protest against the unjust discrimination between applicants for fellowship and the consequent harboring of irregular practitioners, then practiced by the Massachusetts Medical Society, due notice of which protest, after an ineffectual attempt to right matters at home, had been given to the Society by me *viva voce*, at the annual meeting in 1869, and subsequently in print in the *Gynæcological Journal.* This is my first opportunity to bring the matter before the Society. In 1871, at the time of the annual meeting, I was absent in California. In 1872 I was dangerously ill. Since then, and solely in consequence, I have been absent in Europe. Though now residing in Rhode Island, it is for my health alone, and I have refused to apply for, or to accept, membership in its State Society until the stigma that the Councilors have attempted to attach to me shall have been removed.

I now appeal from the Councilors to the Fellows of the Society at large.

HORATIO R. STORER.

Fellow of the Massachusetts Medical Society in full standing.

